# Double common bile duct - A rare case report and review of literature

**DOI:** 10.1016/j.ijscr.2025.111519

**Published:** 2025-06-14

**Authors:** Tu Trong Doan, Duong The Pham, Cuong Van Nguyen, Thanh Tuan Tran, Hai Van Nguyen

**Affiliations:** a Department of Abdominal Surgery 2, Vietnam National Cancer Hospital, Viet Nam; b Department of Radiation Oncology 2, Vietnam National Cancer Hospital, Viet Nam

**Keywords:** Double common bile duct, Duodenopancreatectomy, Colon cancer, Extended right hemicolectomy, Case report

## Abstract

**Introduction:**

Extrahepatic bile duct duplication is an extremely rare congenital anomaly. There are five different types of this malformation. Due to unusual anatomy, the bile duct can be damaged during surgery. We herein report a case of double common bile duct and review the literature about this uncommon anatomical variation.

**Case presentation:**

A 72-year-old female was diagnosed with hepatic flexure colon cancer cT4bN1M0. The tumor invaded the pancreatic head and D2 duodenum in abdomen CT-scan. She underwent an en-bloc extended right hemicolectomy with duodenopancreatectomy and lymph node dissection. During surgery, we discovered that the cystic duct did not open into the common bile duct and there two parallel extrahepatic bile ducts merging into a single duct before draining into the D2 duodenum.

**Discussion:**

Bile duct duplication is an extremely rare congenital variation and has only been reported in the literature as clinical cases. According to Choi, there were five types of these anomalies, our clinical case was classified as type V of this classification.

**Conclusion:**

We presented a case of double common bile duct discovered incidentally during surgery. The surgeons need to carefully expose the extrahepatic biliary tract to avoid bile duct injury.

## Highlights

• Extrahepatic bile duct duplication is an extremely rare congenital anomaly.
• There are five different types of these anatomical variations.
• The surgeons need to expose carefully to discover anomalies of the biliary system to avoid complications.

## 1 Introduction

Bile duct duplication is a rare congenital anomaly, featured by the existence of two parallel extrahepatic bile ducts. The first case of bile duct duplication was presented by Vesalius in 1543, so far there are five different types of these anomalies [1]. Herein we present a patient with bile duct duplication discovered during an extended right hemicolectomy with duodenopancreatectomy due to hepatic flexure colon cancer invading the head of the pancreas and D2 duodenum. Afterward, we will review the literature on this topic. This work has been reported in line with the SCARE criteria [2].

## 2 Case report

A 72-year-old female patient with an insignificant past medical history was admitted to our hospital because of mucus blood stool and dull abdominal pain for the past 3 months. She had no acute severe pain, nausea, or fever. On physical examination, a palpable fixed mass of 6x5cm in size was appreciated on the right upper quadrant. She had no signs of bowel obstruction, anemia, or jaundice. Digital rectal exam revealed no tumor.

**Fig. 1:**
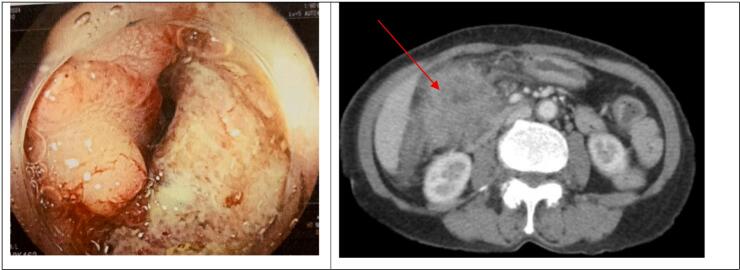
a. An occluding tumor in the hepatic flexure colon in the colonoscopy. b: A tumor 52 × 74 mm in size invaded the pancreatic head and D2 duodenum in the CT scan (red arrow).

Colonoscopy showed imaging of a tumor completely occluding the hepatic flexure colon lumen, the surface of mucosal lesion can easily bleed (Fig. 1a), and a biopsy concluded adenocarcinoma. On gastroscopy, atrophic gastritis was observed. Abdominal CT scan revealed a mass in the hepatic flexure colon of 52x74mm in size, infiltrating D2 of the duodenum, the pancreatic head with some regional lymph nodes (Fig. 1b). No distant metastatic lesion was found on the total body imaging. Routine lab data were normal. CEA level was slightly increased (CEA = 8.1).

**Fig. 2:**
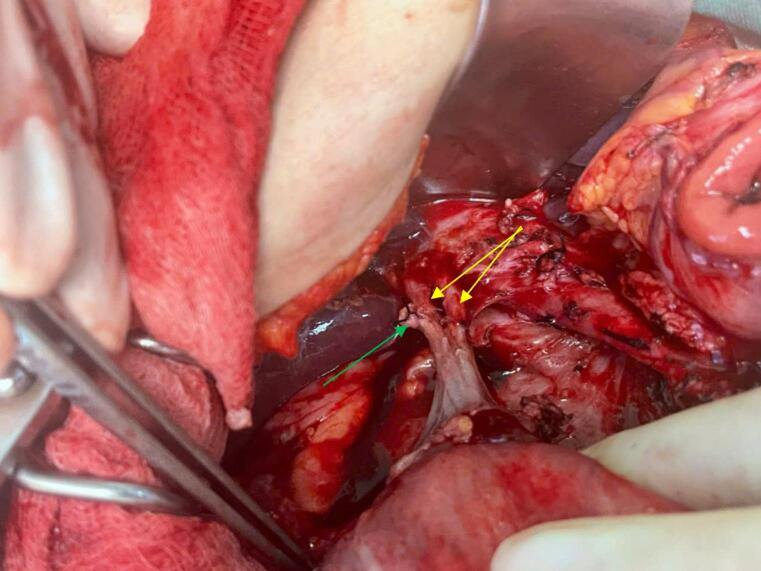
Imaging of extrahepatic bile duct duplication during surgery (yellow arrow: Extrahepatic bile duct duplication, green arrow: cystic duct (ligated)).

The patient was diagnosed with hepatic flexure colon cancer cT4bN1M0 (tumor invaded the head of pancreas and D2 duodenum) and was referred for laparotomy. Intraoperative lesion assessment: The tumor located in the hepatic flexure colon invaded the D2 duodenum and the head of the pancreas, no distant metastasis in the abdomen was observed. We decided to perform an en-bloc extended right hemicolectomy with duodenopancreatectomy and lymph node dissection. During exposing the extrahepatic bile duct, it was assessed that there were two parallel extrahepatic bile ducts that merged into a single duct distally before draining into the D2 duodenum. The cystic drained into the right channel (Fig. 2). After the tumor was removed, we performed 4 anastomotic connections: Ileocolostomy, gastrojejunostomy, pancreaticojejunostomy, and hepaticojejunostomy. Both of the two bile ducts were anastomosed carefully to jejunum by simple interrupted sutures. The total duration of surgery was 5 h and the amount of blood loss was about 200 ml.

Patient's postoperative period was uneventful, drainages were removed 10 days after surgery and she was discharged after 12 days. Final pathology disclosed intermediately differentiated adenocarcinoma of the colon with pancreatic and duodenal tissue invasion, 12/22 positive nodes. She was indicated for adjuvant chemotherapy with XELOX for 8 cycles. There was no recurrence after 9 months of surgery.

## 3 Discussion

The anatomical anomalies of the biliary system are relatively common, however, bile duct duplication is a very rare congenital variation and has only been reported in the literature as clinical cases. The incidence of this variation has not been reported due to its rarity and most individuals are asymptomatic throughout their whole lives, only accidentally discovered during examination or surgery due to other diseases. According to Choi, only 24 cases have been reported in Western countries during the nearly 500 years since the first report by Vesalius in 1543 until 1986 [3]. In Japan, Yamashita compiled 47 reported cases of double bile duct [4]. The mechanism of this anatomical abnormality is unknown. In Boyden's opinion, these abnormalities may be related to the random subdivision of the biliary system during the first week of embryonic development [5].

**Fig. 3:**
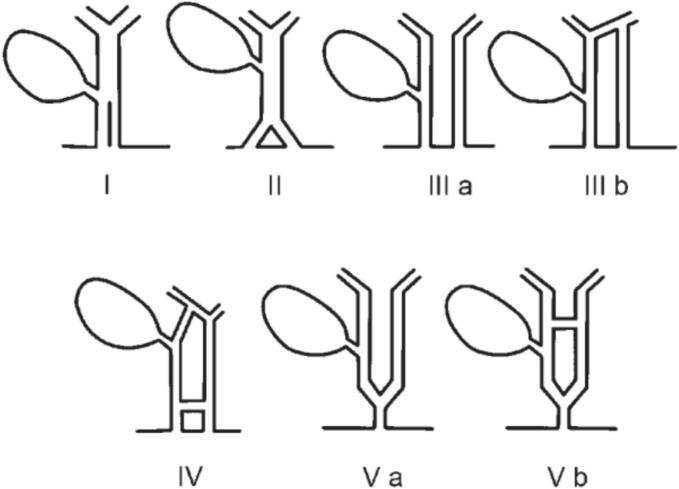
Anatomical anomaly types of bile duct duplication [3].

Based on the anatomical structure, Goor and Ebert were the first authors to classify the variations of double bile duct into 4 types in 1972. In 2007, Choi added one more variant form completing the table of 5 types and this classification is the most popular up to this day (Fig. 3 and Table 1) [3]. Our clinical case was classified as type V, with the characteristic of two bile ducts merging into a common duct before draining into the duodenum. The rate of anatomical variant types also differs according to reports in different regions, for example, the type I variant in China is 58.3 %, which is much higher than in Western countries and Japan, where the rates are 3.5 % and 8.6 % respectively [1].

**Table 1:** Description of bile duct duplication types [3].

Type	Anatomical characteristic
I	Common bile duct with a septum within the lumen
II	Common bile duct which bifurcates with two independent drainages
IIIa	Duplicated extrahepatic bile ducts without intrahepatic communicating duct
IIIb	Duplicated extrahepatic bile ducts with intrahepatic communicating duct
IV	Duplicated extrahepatic bile ducts with extrahepatic communicating channel or both intrahepatic and extrahepatic communicating channels
Va	Duplicated extrahepatic bile ducts join as a single duct and drain into the duodenum without any communication channels
Vb	Duplicated extrahepatic bile ducts join as a single duct and drain into the duodenum with one or more communication channels

Biliary tract duplication is a rare congenital abnormality, so the bile duct can be damaged if the operation is performed by inexperienced surgeons. Hoepfner reported a case of biliary leakage after laparoscopic cholecystectomy due to this anatomical anomaly which was not identified during the operation [6]. This patient underwent reoperation to anastomose both two bile ducts to jejunum. Recognizing this malformation preoperatively can help prevent common bile duct damage during surgery. Endoscopic retrograde cholangiopancreatography (ERCP) is considered as a gold standard in determining the abnormal structure of the bile duct duplication, but it is an invasive intervention and only performed by experienced experts, so it is not routinely indicated. Magnetic resonance cholangiopancreatography (MRCP) is an effective non-invasive method for diagnosis of this abnormality, so it is preferred prior to ERCP. MRCP should be considered before complex surgeries involving the biliary system. However, many cases of bile duct duplication are difficult to identify in preoperative investigation and are only detected incidentally during surgery. The anomaly type I is the most challenging to detect, only 4.2 % of cases can diagnosed preoperatively [7].

Comorbidities along with bile duct duplication are diverse. Yamashita summarized 47 cases of bile duct duplication that were published in Japan. Among these, the rate of cholelithiasis was 27.7 %, choledochal cyst was 10.6 %, pancreaticobiliary maljunction was 29.8 %, and cancers occur in 25.5 % of cases, primarily gastric cancer, pancreatic cancer, ampullary cancer, and gallbladder cancer [4]. Double bile duct abnormalities are believed to be related to the risk of cancer. Yamashita emphasized the clinical importance of the opening site of the double common bile duct rather than its anatomic appearance. Gastric cancer usually appears in patients with bile duct opening into the stomach, while most of biliary system cancers are associated with pancreaticobiliary maljunction [8].

## 4 Conclusion

In conclusion, we presented a case of extrahepatic bile duct duplication discovered incidentally during surgery. The surgeon needs to carefully distinguish the anatomy of the biliary system during the dissection to prevent bile duct injury.

## CRediT authorship contribution statement

Tu Trong Doan: the main doctor conceived the original idea and operated the patient, revised manuscript.
Duong The Pham: operated the patient, followed up, wrote manuscript.
Cuong Van Nguyen: operated the patient, followed up, revised manuscript.
Thanh Tuan Tran: followed up, revised manuscript.
Hai Van Nguyen: followed up, revised manuscript.

## Consent

Written informed consent was obtained from the patient for publication of this case report and accompanying images. A copy of the written consent is available for review by the Editor-in-Chief of this journal on request.

## Ethical approval

The patient is anonymized and does not include any identifiable personal information. The manuscript was approved by ethical committee of Viet Nam National cancer hospital.

## Guarantor

Tu Trong Doan, MD.

## Research registration number

Not applicable.

## Funding

The authors received no financial support for the research, authorship, and/or publication of this report.

## Declaration of competing interest

The authors declare that they have no competing interests relevant to the content of this article.
